# Orbital involvement by NUT midline carcinoma: new presentation and encouraging outcome managed by radiotherapy combined with tyrosine kinase inhibitor: a case report

**DOI:** 10.1186/s13000-019-0922-1

**Published:** 2020-01-04

**Authors:** Peiwei Chai, Chuandi Zhou, Renbing Jia, Yefei Wang

**Affiliations:** 1 Department of Ophthalmology, Ninth People’s Hospital, Shanghai JiaoTong University School of Medicine, Shanghai, 200025 People’s Republic of China; 2 Shanghai Key Laboratory of Orbital Diseases and Ocular Oncology, Shanghai, China

**Keywords:** NUT midline carcinoma, Epiphora, Anlotinib, Tyrosine kinase inhibitor

## Abstract

**Background:**

NUT midline carcinoma (NMC) is a poorly differentiated squamous cancer with a median survival at less than 7 months. NMC is resistant to conventional chemotherapies and characterized by rearrangement of the *NUT* gene.

**Case presentation:**

Here, we described a patient who initially presented with epiphora, and an orbit involved NMC. In addition, we for the first time demonstrated that local radiotherapy combined with tyrosine kinase inhibitor **(**TKI) could significantly inhibit tumor progression in orbital involvement by NMC.

**Conclusions:**

Our study for the first time described an orbit involved NMC patient initially presented with epiphora. In addition, we provided an alternative to the management of orbit involved NMC.

## Background

NUT midline carcinoma (NMC) is a rare tumor arising primarily in the midline with a median survival at less than 7 months [1,2]. NMC is resistant to conventional chemotherapies and characterized by rearrangement of the *NUT* gene [3]. To our best knowledge, no report has documented an NMC with primary presentation of epiphora. Due to the rapid progression of NMC, the patient missed the best chance of surgical resection. Notably, the recent availability of targeted therapy with Bromo- and Extra-Terminal domain inhibitors (BETi) may hold promise in treating NMC. However, BETi is not currently available in China. In this report, we performed local radiotherapy with Anlotinib, which significantly inhibited NMC progression and could provide an example of an alternative therapeutic option for NMC.

## Case presentation

**Fig. 1:**
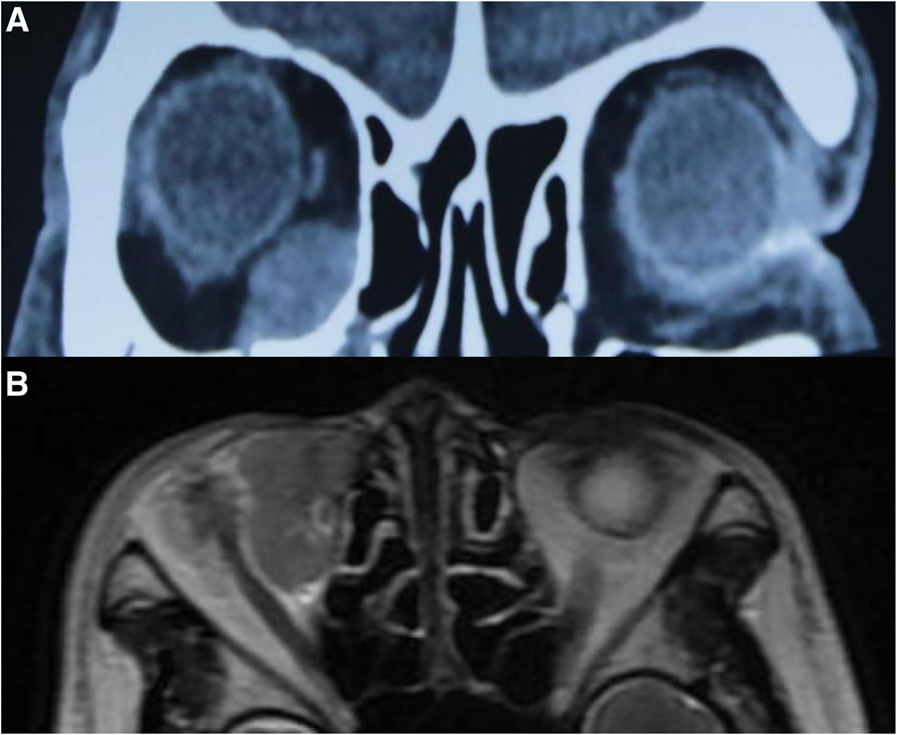
An orbit involved NUT midline carcinoma (NMC). **a** The orbital computed tomography (CT) and (**b**) Magnetic resonance imaging (MRI) showed an orbital mass extending to the adjacent paranasal sinuses

**Fig. 2:**
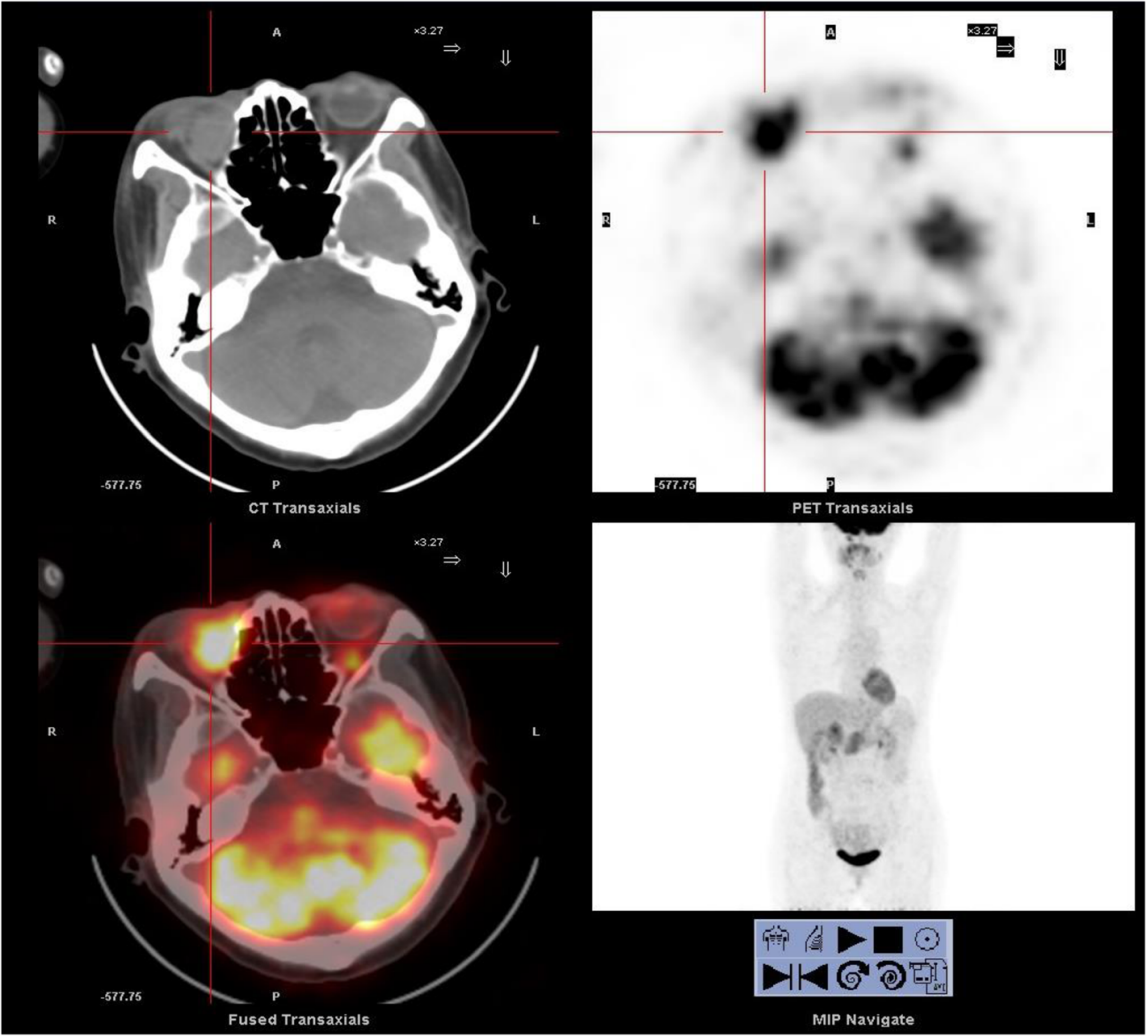
A systemic ^18^F-2-Fluoro-2-Deoxy-D-Glucopyranose positron emission tomography (^18^F-FDG PET/CT) demonstrated an orbital mass with increased FDG uptake but no other remarkable malignancy in the trunk

**Fig. 3:**
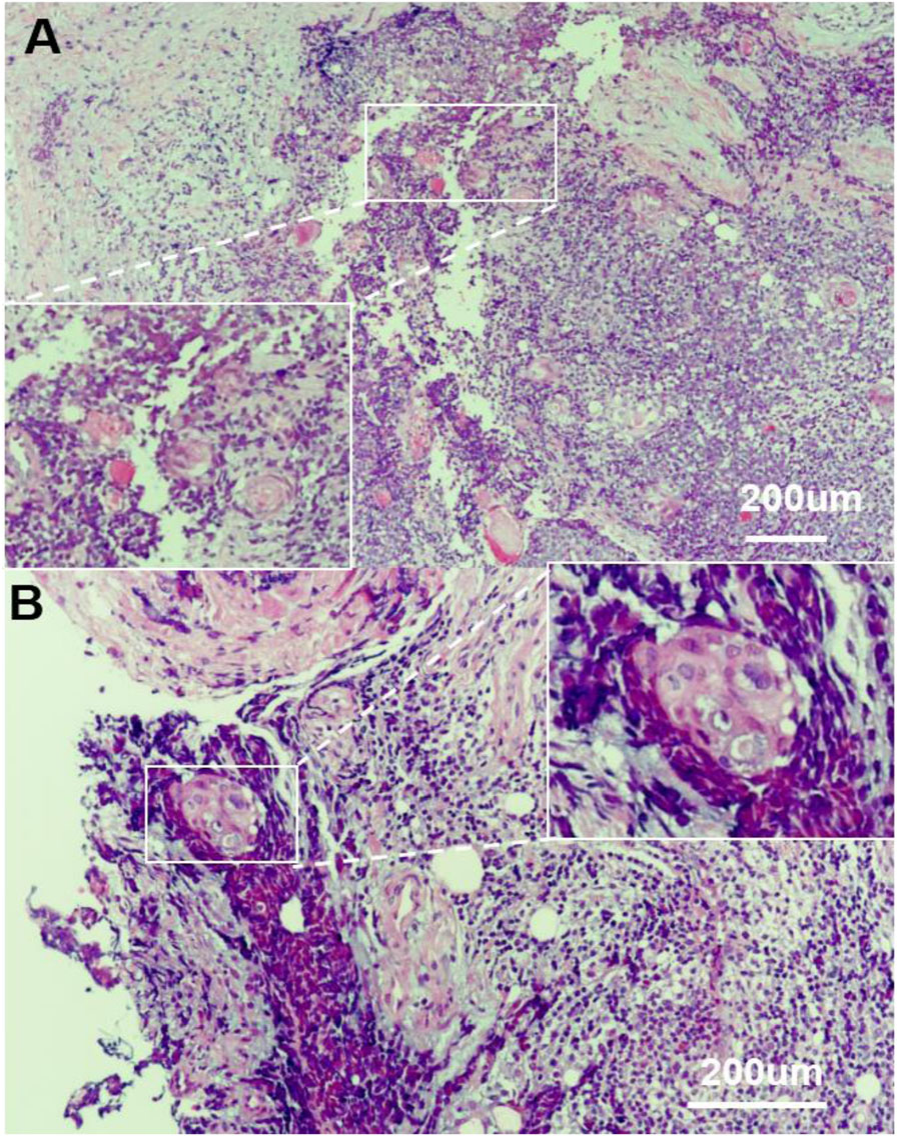
Hematoxylin-eosin (HE) staining demonstrated a NUT midline carcinoma with aberrant squamous differentiation. **a**-**b**: A malignant epithelial neoplasm with squamous features with direct juxtaposition of basaloid, immature and undifferentiated squamous cells. Scale bar: 50um

**Fig. 4:**
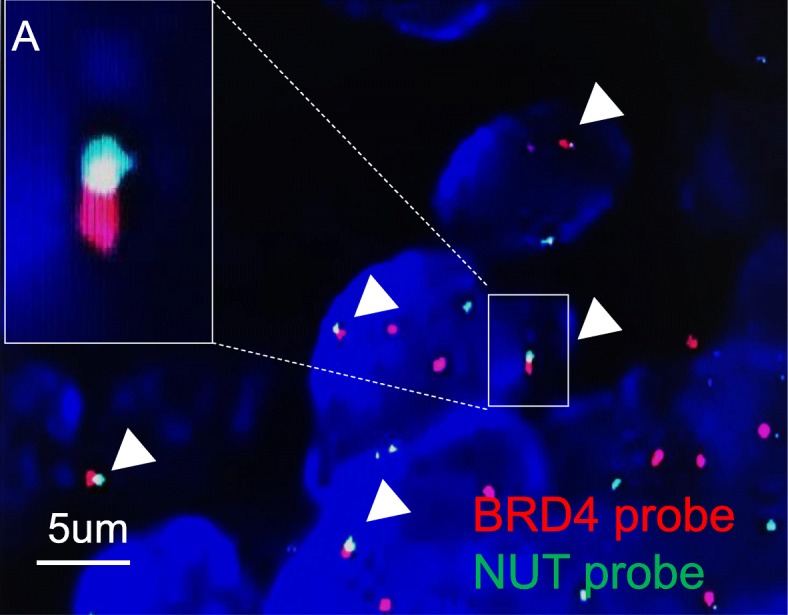
Fluorescent DNA in situ hybridization (FISH) demonstrated a *BRD4-NUT* rearrangement (White triangle). A red probe that spans *NUT* splits and joins the green *BRD4* centromeric probe. Scale bar: 5um

**Fig. 5:**
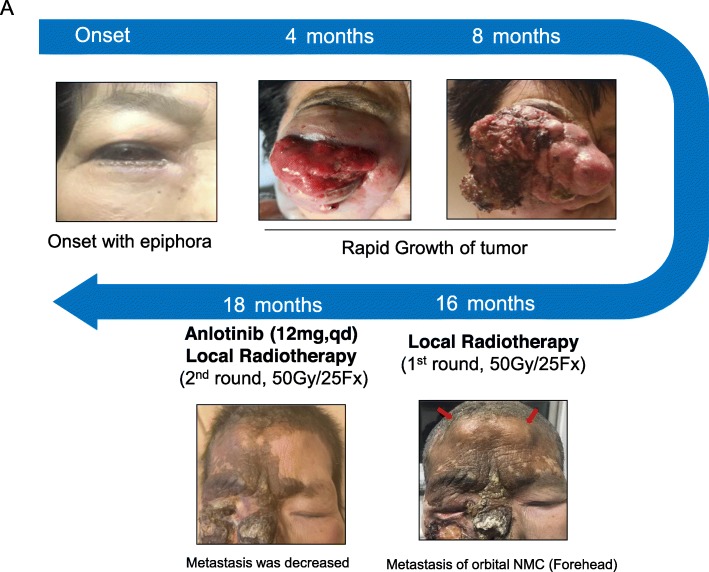
The appearance of the NUT midline carcinoma (NMC) patient at the time of onset, 4, 8, 16 and 18 months after diagnosis. The patient’s consent for using their photos for publication has been obtained

A 59-year-old female presented with 14 days of epiphora. She denied vision loss, pain, epistaxis, or fevers. Her complete blood count examination showed Hb 112 g/L [ref. 113~151 g/L], WBCs 4.2 × 10^9^/L [ref. 4.0~10.0 × 10^9^/L], and Platelets 213 × 10^9^/L [ref. 100~300 × 10^9^/L]. Her renal function test (RFL), liver function test (LFT) and lipid profile were within normal limits (DBIL 2.0 μmol/L [ref. 0.51~3.42umol/L], I-BIL 2.4 μmol/L [ref. 1.71~13.8umol/L], Scr 66 μmol/L [ref. 30~110umol/L], BUN 3 mmol/L [ref. 2.9~7.5 mmol/L] and LDL 2.78 mmol/L [ref. 1.9~3.6 mmol/L]). Irrigation of the lacrimal passage suggested no blockage, no purulent or hemorrhagic discharge. Three months later, the symptom of epiphora aggravated. Orbital computed tomography (CT) and magnetic resonance imaging (MRI) scans showed a right orbital mass extending to the adjacent paranasal sinuses (Fig. 1). The results of a gastrointestinal tract endoscopy, colonoscopy, nasal endoscope and ^18^F-2-Fluoro-2-Deoxy-D-Glucopyranose positron emission tomography (^18^F-FDG PET/CT) revealed no other malignancies (Fig. 2). The mass was surgically removed. Pathologic analysis suggested a malignant epithelial neoplasm with squamous features with direct juxtaposition of basaloid, immature and undifferentiated squamous cells (Fig. 3). A panel of immunohistochemistry stains showed positive staining for markers of squamous differentiation, for p40(+), p63(+), CK5/6(+), NUT (+) and Ki67(50%+). Fluorescent DNA in situ hybridization (FISH) demonstrated the presence of *NUT-BRD4* rearrangement (Fig. 4). In this condition, orbital exenteration was indicated, however, the patient refused. The mass grew rapidly after primary resection, which metastasized to cervical lymph node 2 months later (pathologically proved with biopsy). The patient developed severe dyspnea and could hardly perform prostrations (Fig. 5, 8-month). 4 months later, the patient was treated with first round of local radiotherapy (50 Gy/25 Fx), tumors shrunk, and the symptom of dyspnea eased. No remarkable adverse effects were observed. However, 2 months later, more metastasis was observed in the forehead and neck (Fig. 5, red arrow). The patient was then treated with multi-targeting tyrosine kinase inhibitor (Anlotinib, 12 mg, qd) and second round of local radiotherapy (50 Gy/25 Fx) thereafter. The masses continued to shrink, and the lymph node metastasis was significantly decreased (Fig. 5, 18-month). Except for gingival bleeding, no other serious adverse effects have been observed. To date, the patient had an 8-month disease-free survival.

## Discussion and conclusions

NMC is an aggressive subtype of squamous cell carcinoma, which is caused by chromosomal rearrangement of the gene nuclear protein in testis (*NUT*) on 15q14 [4,5]. In 80% of NMC patients, the chromosomal translocation occurs between the *NUT* and *BRD4* genes, leading to the occurrence of a *BRD4-NUT* fusion gene [6]. To date, a standard treatment for NMC has not been established and a multi-target treatment strategy with systemic chemotherapy, surgery and radiation therapy is currently adopted in clinical practice [7]. For this patient, *BRD4-NUT* encoded fusion proteins, which block epithelial cell differentiation and contribute to carcinogenesis, were examined by FISH assay. Although NMC remains an underrecognized squamous carcinoma, there has appeared to be increasing awareness of disease and frequency of diagnosis [8]. Typically, NMC arises from midline supradiaphragmatic structures: the upper aerodigestive tract (50%) and the mediastinum (41%). Moreover, rarer cases have been observed below the diaphragm (bladder) and outside the midline axis (major salivary glands, iliac bone, adrenal gland, and pancreas). Here, we for the first time revealed that the NMC, initially presented with epiphora.

Previous studies have presented grave prognoses for NMC, with a limited life expectancy and a > 80% chance of death within the first year of diagnosis [9]. Additionally, aggressive surgical resection and early radiotherapy may prolong life expectancy for the non-metastatic NMC patient [4]. In this case, we assumed that the NMC grew rapidly after primary resection was due to the incomplete resection. And the patient refused orbital exenteration for disfigurement of facial appearance. The recent availability of targeted therapy with acetyl-histone mimics (BETi) may hold promise in treating NMC [2]. Jeremy Lewin et al. found that NMC had a partial response with 1.4 to 8.4 months of BETi treatment [10]. However, BETi is not currently available in China. In this report, we performed local radiotherapy combined with multi-targeting tyrosine kinase inhibitor (Anlotinib) could significantly suppress NMC progression, which might provide an alternative to the treatment of NMC.

Receptor tyrosine kinases (RTKs) are transmembrane glycoproteins that communicate with cellular growth factors and extracellular ligands [11]. They play vital roles in intracellular tyrosine phosphorylation and intracellular signaling [12]. RTK activation mediates many vital physiological processes, including proliferation, migration, differentiation and apoptosis [13]. In addition, RTKs have been implicated in a variety of cancers, such as non-small-cell lung cancer (NSCLC), renal cell carcinoma (RCC) and advanced medullary thyroid cancer [13]. Anlotinib is a novel, orally administered RTKs inhibitor that multi-targets fibroblast growth factor receptor (FGFR), vascular endothelial growth factor receptor (VEGFR) and platelet-derived growth factor receptors (PDGFR) [14]. Compared to placebo, it largely improved both progression-free survival (PFS) and overall survival (OS) in a phase III trial in patients with advanced non-small-cell lung cancer (NSCLC) [15]. Here, we for the first time demonstrated that the clinical application of Anlotinib in treating with orbital NMC.

In summary, we initially reported a case of NMC with the primary complaint of epiphora. In addition to routine tests of lacrimal duct irrigation, a CT or MRI should be carried out if the symptoms persist. Heightened awareness and early recognition of NMC are critical, and the clinician should possess adequate suspicion for NMC, especially when encountering patients with refractory epiphora. NMC is histopathologically characterized by abrupt squamous differentiation, a NUT immunostaining positive result and *BRD4-NUT* rearrangement by FISH assay. Once diagnosed with NMC, complete surgical resection should be performed. Adequate radiotherapy is recommended postoperatively. Additionally, other adjuvant therapies, such as BETi, local radiotherapy and tyrosine kinase inhibitor, could also be considered for NMC treatment. Larger studies and longer follow up time still needed to validate our finding.

## Data Availability

N.A.

## Abbreviations

BETi: Bromo- and Extra-Terminal domain inhibitors
CT: Computed tomography
FGFR: Fibroblast growth factor receptor
FISH: Fluorescent DNA in situ hybridization
LFT: Liver function test
MRI: Magnetic resonance imaging
NMC: NUT midline carcinoma
NUT: Nuclear protein in testis
PDGFR: Platelet-derived growth factor receptors
PET: Positron emission tomography
RFL: Renal function test
RTKs: Receptor tyrosine kinases
VEGFR: Vascular endothelial growth factor receptor
